# Quantum coupled mutation finder: predicting functionally or structurally important sites in proteins using quantum Jensen-Shannon divergence and CUDA programming

**DOI:** 10.1186/1471-2105-15-96

**Published:** 2014-04-03

**Authors:** Mehmet Gültas, Güncel Düzgün, Sebastian Herzog, Sven Joachim Jäger, Cornelia Meckbach, Edgar Wingender, Stephan Waack

**Affiliations:** 1 Institute of Computer Science, University of Göttingen, Goldschmidtstr. 7, 37077 Göttingen, Germany; 2 Institute of Bioinformatics, University of Göttingen, Goldschmidtstr. 1, 37077 Göttingen, Germany

## Abstract

**Background:**

The identification of functionally or structurally important non-conserved residue sites in protein MSAs is an important challenge for understanding the structural basis and molecular mechanism of protein functions. Despite the rich literature on compensatory mutations as well as sequence conservation analysis for the detection of those important residues, previous methods often rely on classical information-theoretic measures. However, these measures usually do not take into account dis/similarities of amino acids which are likely to be crucial for those residues. In this study, we present a new method, the Quantum Coupled Mutation Finder (QCMF) that incorporates significant dis/similar amino acid pair signals in the prediction of functionally or structurally important sites.

**Results:**

The result of this study is twofold. First, using the essential sites of two human proteins, namely epidermal growth factor receptor (EGFR) and glucokinase (GCK), we tested the QCMF-method. The QCMF includes two metrics based on quantum Jensen-Shannon divergence to measure both sequence conservation and compensatory mutations. We found that the QCMF reaches an improved performance in identifying essential sites from MSAs of both proteins with a significantly higher Matthews correlation coefficient (MCC) value in comparison to previous methods. Second, using a data set of 153 proteins, we made a pairwise comparison between QCMF and three conventional methods. This comparison study strongly suggests that QCMF complements the conventional methods for the identification of correlated mutations in MSAs.

**Conclusions:**

QCMF utilizes the notion of entanglement, which is a major resource of quantum information, to model significant dissimilar and similar amino acid pair signals in the detection of functionally or structurally important sites. Our results suggest that on the one hand QCMF significantly outperforms the previous method, which mainly focuses on dissimilar amino acid signals, to detect essential sites in proteins. On the other hand, it is complementary to the existing methods for the identification of correlated mutations. The method of QCMF is computationally intensive. To ensure a feasible computation time of the QCMF’s algorithm, we leveraged Compute Unified Device Architecture (CUDA).

The QCMF server is freely accessible at http://qcmf.informatik.uni-goettingen.de/.

## Background

Multiple sequence alignments (MSAs) of homologous protein sequences give us information about two major features of the proteins of interest. The first one consists of easily detectable highly conserved residue sites that are obviously important for the structure and/or the function of the protein; while the second one corresponds to compensatory (coupled) mutations between two or more residue sites that also contain crucial information on the structural and functional basis of proteins [1]. These compensatory mutations occur according to the functional coupling of mutation positions which might be explained as one mutation in a certain site affecting a compensating mutation at another site, even if both related residue sites are distantly positioned in the protein structure [2-5]. In particular, such mutations at essential residue sites are likely to destroy protein structure which often results in loss of the protein function [6,7]. Thus, recognition of these residue sites is as important as the strictly conserved positions for the understanding of the structural basis of protein functions and for the identification of functionally important residue positions [5,8,9].

Although the strictly conserved residue sites are easily detectable and interpretable in MSAs, the detection of important non-conserved compensatory mutation sites needs more complex approaches. Today, due to the simplicity and efficiency, the mutual-information-based metrics (MI-metrics) are often used to measure the co-evolutionary relationship between residue sites in MSAs [4-6,10-13]. However, the MI-metrics strongly depend on the amino acid distributions observed in the MSA columns rather than on physical or biochemical constraints of amino acids that are likely to be crucial for the detection of functionally or structurally important compensatory mutations in a protein sequence. Further, according to the phylogenetic relationship of protein sequences and background noise, there is always a MI-value between each column pair in an MSA. Therefore, the challenging problems in bioinformatics for the detection of significant compensatory mutation signals are: i) the minimization of the influence of phylogenetic relationships of protein sequences by incorporating physical or biochemical properties of amino acids in the calculation; ii) the separation of significant signals from the background noise or unrelated pair signals.

In order to eliminate the influence of phylogeny and noise effects of MI, Dunn et al. [6] have introduced the average product correction (APC). Subtracting APC from MI, they obtained their MIp metric. However, in their model the reduction of background noise is not quantified. On the other hand, Gao et al. [13] have integrated amino acid background distribution (MIB) in the calculation of their MI-metric and focused on only 25 column pairs of each MSA with the highest normalized MI values as significant to reduce noisy effect which seems to be over-conservative, yet specific.

Large efforts have been made in the last few years to improve local-correlation-measure-based approaches to residue co-evolution when it comes to modeling effects that rely on spatial proximity (see [14] for an overview). In this case, it is necessary to disentangle direct and indirect correlations. Classical mutual information, for example, is high not only if the two sites under study are close in 3D space. Quite the contrary, any local measure of correlation, not just mutual information, is limited by the transitivity effect.

To overcome this problem, global statistical models of protein families are employed. The direct-coupling analysis (DCA) works as follows. Maximizing the entropy subject to preserving the single and pair residue frequencies observed, a joint probability distribution on all possible members of the protein family is derived. Utilizing this distribution, considerable progress in predicting residue-residue contacts in 3-dimensional protein structures was made [15-17]. Protein Sparse Inverse Covariance (PSICOV) [18] achieves disentanglement of direct and indirect correlations by inverting a residue-residue covariance matrix. In [19] further progress was made by integrating structural context and sequence co-evolution information.

There is merely a small number of methods that incorporate amino acid similarity in the prediction of functionally or structurally important sites. In this context, it is natural to partition the amino acids into chemically similar groups before applying an information-theoretic measure like the Shannon entropy [20,21]. It was reported that many other methods fail to outperform this simple partition approach [22]. However, quantum information theory supplies a well-studied and powerful framework to integrate such similarity, where the classical Shannon entropy is swapped for the von Neumann entropy (VNE). Caffrey et al. [23] and Johansson et al. [24] have firstly introduced VNE to multiple sequence alignment analysis although they did not treat amino acid pair similarity.

Recently, a new method called Coupled Mutation Finder (CMF) has been introduced by Gültas et al. [5] to deal with phylogenetic noise as well as background signals and to quantify the error made in terms of the false discovery rate. The CMF method only focuses on BLOSUM62-dissimilar amino acid pairs as a model of compensatory mutations and integrated them in the calculation of normalized MI-metrics using a doubly stochastic matrix to transform the empirical pair distribution of the column pair. However, the CMF disregards amino acid pair similarity which can be also crucial for the detection of functionally or structurally important sites in MSAs.

In this study, we present a new method called Quantum Coupled Mutation Finder (QCMF) which extends the CMF algorithm [5] by additionally incorporating amino acid pair similarity. To this end, the QCMF invokes principles from quantum information theory, in particular for the first time in the context of MSA analysis quantum entanglement as a major resource of quantum information. Amino acid pair distributions are replaced by entangled density matrices from quantum mechanics which encompass in our case both empirical pair distributions, possibly transformed by the doubly stochastic matrix used in [5], and pair similarity. Following Capra and Singh [22] who pointed out that it is hard to improve upon metrics based on Jensen-Shannon divergences, we quantify the effect of both amino acid pair similarity and amino acid pair dissimilarity by the quantum Jensen-Shannon divergence between an entangled density matrix and the one that simply represents the amino acid pair frequencies.

The QCMF algorithm is strongly based on the matrix operations that are computationally intensive. When analyzing a single MSA, the computational time of these matrix operations rise very quickly due to the huge number of column pairs. In order to speed up the running time of the QCMF, we implemented its algorithm using Compute Unified Device Architecture (CUDA). CUDA is an efficient parallel computing architecture developed by NVIDIA that utilizes graphic processing units (GPUs) for general-purpose scientific and engineering applications [25]. Nowadays, GPUs are often used for computationally challenging problems in bioinformatics [26-29] and several other scientific fields [30-32].

## Results

Our main focus in this study was to investigate whether quantum information theory based measures could contribute beyond conventional measures to the identification of important residue sites. The Results section of this work twofold. First, to test the functionality of QCMF-significant individual residue sites we analysed the essential sites of two human proteins: epidermal growth factor receptor (EGFR) (pdb entry 2J6M) and glucokinase (GCK) (pdb entry 1V4S). The functionally and structurally important sites of both proteins have been experimentally investigated in several studies previously [33-44] and their positions were summarized in [5] as essential sites. The essential sites of these proteins consist of several non-conserved residue sites which are directly located at or near disease associated amino acid mutation (non-synonymous single nucleotide polymorphisms (nsSNPs)) sites, catalytic sites, protein binding sites and so on, each of which are likely to affect protein stability or functionality (see [5] and references therein). In addition, residue sites are defined to be in contact according to the “nearby” definition of Nussinov et al. [45] if their carbon major atoms have a distance of less than or equal to 6 Å. Consequently, we defined an individual QCMF-significant residue site as “functionally or structurally important” if it corresponds to one of these essential sites.

Second, to further investigate the performance of QCMF and to make a comparison with the previous methods (CMF [5], MIp [6], and PSICOV [18]), we selected a non-redundant set of proteins prepared by Janda et al. [46]. Although the dataset contains 216 proteins, we eliminated a few proteins due to inconsistency between corresponding MSAs and PDB files, so that we finally ended up with a dataset of 153 proteins (see Additional file 1).

The MSAs for each protein, which contain after filtering at least 125 independent sequences, were derived from the HSSP-database [47] that merges primary structure information and tertiary structure information of proteins.

Finally, we define QCMF-significant sites as follows. Let *M* be an MSA, with the protein of interest being the first row of *M*. A site pair as well as an individual site of the protein are said to be QCMF-significant with respect to the MSA *M*, if they are $\left({\mathbb{Q}}_{\text{ent}},M\right)$-significant or $\left({\mathbb{Q}}_{\text{sep}},M\right)$-significant. The latter two notions and the underlying two co-evolutionary column pair metrics ${\mathbb{Q}}_{\text{ent}}$ and ${\mathbb{Q}}_{\text{sep}}$ are defined in the Methods section. If the MSA *M* is fixed, we speak of ${\mathbb{Q}}_{\text{ent}}$-significance and ${\mathbb{Q}}_{\text{sep}}$-significance, rather than of $\left({\mathbb{Q}}_{\text{ent}},M\right)$-significance and $\left({\mathbb{Q}}_{\text{sep}},M\right)$-significance, respectively.

### 

#### QCMF-significant residue sites in the Human Epidermal Growth Factor Receptor (EGFR) protein

Using the MSA-specific statistical model with a false discovery rate (FDR) of 1% for both QCMF-metrics, we first determined altogether 2688 out of 26079 non-conserved column pairs as significant in corresponding MSA of human EGFR protein. 631 of these significant pairs were detected by ${\mathbb{Q}}_{\text{ent}}$-metric, and 2149 pairs were detected by ${\mathbb{Q}}_{\text{sep}}$-metric. Only 92 significant column pairs were detected by both metrics. After that, utilizing the connectivity degree technique, we predicted in total 33 residue sites in corresponding sequence of human EGFR protein as QCMF-significant (see Additional file 2). 12 of them are only ${\mathbb{Q}}_{\text{ent}}$-significant and 18 residue sites are ${\mathbb{Q}}_{\text{sep}}$-significant, the remaining 3 residue sites (A839, A882 and V902) are both ${\mathbb{Q}}_{\text{ent}}$-significant and ${\mathbb{Q}}_{\text{sep}}$-significant.

**Figure 1:**
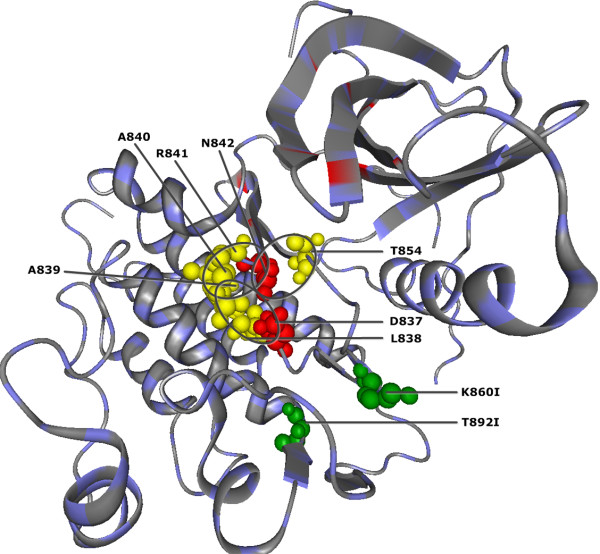
**QCMF-significant residue positions are in contact with catalytic residues in human EGFR protein (PDB-Entry 2J6M).** Red spheres denote positions of the catalytic residues. Yellow spheres show the localization of significant adjacent residue positions found by QCMF which are in contact with these catalytic residues. Moreover, the QCMF-significant sites A839 and R841 are also catalytic residues by themselves. Green spheres show the structural localization of nsSNP positions found by QCMF as significant in the EGFR protein. The circles indicate clusters of catalytic residue sites and their significant adjacent sites.

**Figure 2:**
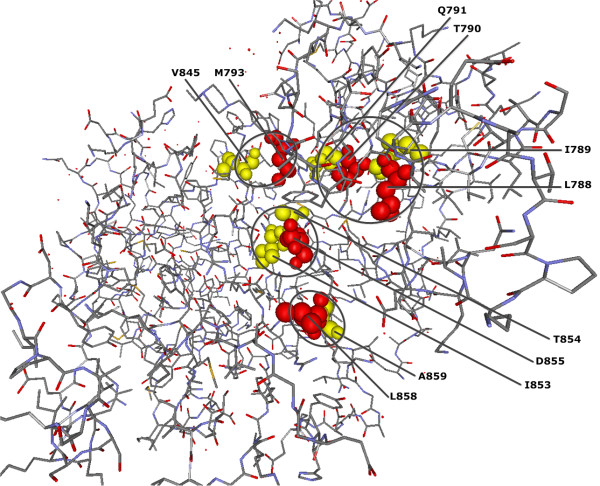
**QCMF-significant residue positions are in contact with gefitinib binding sites in human EGFR protein (PDB-Entry 2J6M).** Red spheres show the structural localization of the gefitinib binding sites in the wild type kinase. Yellow spheres show QCMF-significant adjacent residue positions which are in contact with these binding sites. Moreover, the QCMF-significant site T854 is also a binding site by itself and interacts with gefitinib binding site D855. The circles indicate clusters of gefitinib binding sites and their significant adjacent sites.

10 of the QCMF-significant residue sites are in contact with either catalytic residues or critical active site regions for gefitinib binding site in wild type EGFR kinase [34,37,48] (see Figure 1 and Figure 2). Among these sites, the A839 and R841 have been verified as catalytic residue sites through the Catalytic Site Atlas [48]. The T854 is a gefitinib binding site by itself and the residue sites V845 and A859 are also in contact with nsSNP positions K846, T847 and K860 in human EGFR protein. Moreover, two out of all 33 significant sites are related to disease associated nsSNP positions and their structural localization are illustrated in Figure 1.

Additionally, 13 out of all QCMF-significant sites are referred to as essential sites, each of them are either nearby strictly conserved residues or nsSNPs (see Table 1).

**Table 1:** QCMF-significant essential sites in the human EGFR protein, which are nearby either nsSNPs or strictly conserved sites

**QCMF-significant**	**Nearby nsSNPs, or strictly**	**Reference**
**essential sites**	**conserved sites**	
*N*771	773^**s**^	[44]
*G*824	773^**s**^	[44]
*Y*827	829^**s**^	[44]
*L*828	829^**s**^	[44]
*V*834	835^**c**^, 836^**s**^,860^**s**^	[44,49]
*Y*891	892^**s**^, 895^**c**^	[44]
*A*822	861^**s**^	[43,49,50]
*V*844	796^**c**^, 798^**c**^, 852^**c**^	-
*A*882	884^**c**^, 895^**c**^, 898^**c**^	-
*Y*900	898^**c**^, 901^**c**^	-
*V*902	880^**c**^, 901^**c**^	-
*T*909	906^**c**^, 936^**c**^	-
*G*911	906^**c**^	-

^**s**^ : non-synonymous snp site, ^**c**^ : strictly conserved site.

According to the essential sites of human EGFR protein, published in [5], we have shown altogether the structural or functional importance of 25 QCMF-significant sites. The remaining 8 significant residue sites (G729, T851, G779, Q820, M825, L927, G930, Y944) do not fall into essential sites and the reason for their significance and their importance in the EGFR protein is currently unclear.

#### **QCMF-significant residue sites in the Human Glucokinase (GCK) protein**

Like human EGFR protein, applying the MSA-specific statistical model with a FDR of 1% for both QCMF-metrics we identified a total of 9853 out of 69645 non-conserved column pairs as significant in the human GCK protein (pdb entry 1V4S). 6070 of them were $\left({\mathbb{Q}}_{\text{ent}},M\right)$-significant and 4232 were detected as $\left({\mathbb{Q}}_{\text{sep}},M\right)$-significant. Only 449 column pairs were detected as significant with respect to both metrics. Thereupon using the connectivity degree technique, we determined altogether 64 residue sites in the human GCK protein as QCMF-significant (see Additional file 3). 30 of them are determined as ${\mathbb{Q}}_{\text{ent}}$-significant and further 30 significant residue sites are determined as ${\mathbb{Q}}_{\text{sep}}$-significant. Only four residue sites (T82, G223, V253, and G407) are significant based on both metrics.

13 of QCMF-significant sites are in contact with allosteric sites V62, R63, M210, I211, Y214, Y215, M235, V452, V455 and A456 in the human GCK protein. Among these significant sites, the *V*62, *M*210, *Y*215 are allosteric sites by themselves [41] and the T209M, G223S and S453del are related to disease associated nsSNP positions. In addition, there are further five QCMF-significant sites (F123L, G162D, G175R, T228M, and E300K,Q) that have been verified as nsSNP positions through annotation databases and previous experimental studies [38-40,42,43,51]. The structural localization of these 18 QCMF-significant sites (contact sites and nsSNPs positions) are illustrated inFigure 3.

**Figure 3:**
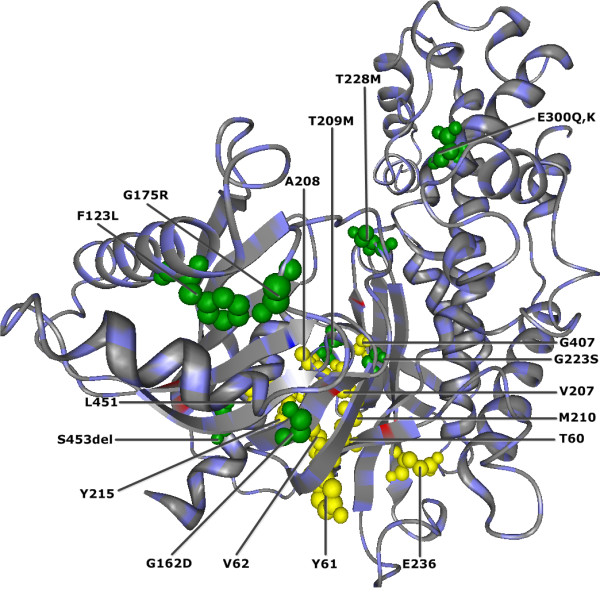
**QCMF-significant positions that are either in contact with allosteric sites or related to nsSNPs in human GCK protein (PDB-Entry 1V4S).** Yellow spheres correspond to structural localization of ten significant residue sites which are in contact with allosteric sites where V62, M210, and Y215 are denoted as allosteric sites by themselves and they are also in contact with an other allosteric sites. Green spheres indicate eight significant nsSNP positions in the GCK protein. Three of them (T209M, G223S and S453del) are further in contact with allosteric sites M210, I211, V452, V455 and A456.

**Figure 4:**
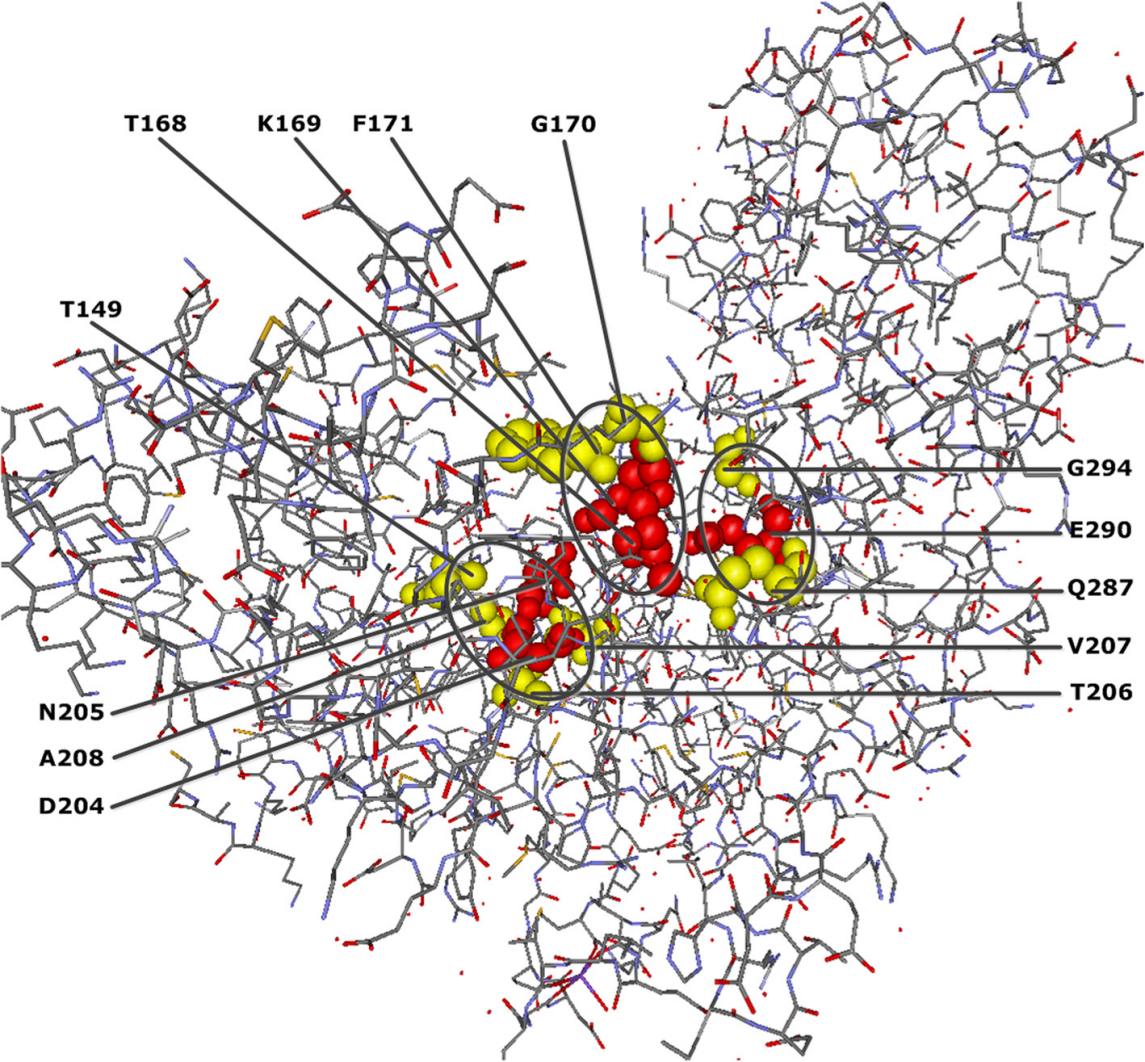
**QCMF-significant residue positions are in contact with glucose binding site in human GCK protein (PDB-Entry 1V4S).** (A) Red spheres show the structural positions of the glucose binding sites (active sites) and yellow spheres show the localization of significant adjacent residue positions found by QCMF which are in contact with these active sites. The circles indicate clusters of glucose binding sites and their significant adjacent sites.

Additionally, eight significant sites T149, G170, F171, T206, V207, A208, Q287 and G294 in contact with glucose binding sites (active sites) T168, K169, D204, D205 and E290 in human GCK protein [41] (see Figure 4) where V207 and A208 are also in contact with the allosteric sites M210 and I211.

Moreover, we have also observed that 38 QCMF-significant sites are further included in essential sites since they are nearby nsSNPs or strictly conserved residues in human GCK protein (see Table 2).

**Table 2:** QCMF-significant essential sites in the human GCK protein, which are nearby either nsSNPs or strictly conserved sites

**QCMF-significant**	**Nearby nsSNPs or strictly**	**Reference**
**essential sites**	**conserved sites**	
*M*37	36^**s**^,39^**s**^,40^**s**^	[38,39,43,51]
*S*76	147^**c**^	
*L*79	78^**c**^,80^**c**^,150^**c**^	-
*T*82	81^**c**^	-
*N*83	81^**c**^,108^**s**^,110^**s**^	[43,51]
*V*86	85^**c**^,106^**s**^	[38]
*S*127	130^**s**^	[40]
*F*148	147^**c**^,150^**c,s**^	[38,39,43,51]
*F*152	150^**c,s**^, 151^**c**^	[39,43,51]
*P*153	154^**s**^	[39]
*H*156	154^**s**^	[39]
*A*176	119^**s**^,175^**s**^	[43]
*G*178	164^**c**^	
*N*180	162^**s**^,182^**s**^	[38,39,43],
*L*185	182^**s**^,188^**s**^	[39,43,51]
*A*201	147^**c**^,453^**c**^	
*M*202	147^**c**^,203^**s**^	[43]
*A*232	223^**s**^,231^**c**^	[39,40,51]
*C*233	223^**s**^,234^**c**^,235^**c**^	[39,40,51]
*V*253	234^**c**^,254^**c**^	
*F*260	257^**s**^,258^**c**^,259^**s**^,261^**s**^	[39,43]
*L*271	274^**c**^	
*V*277	274^**c**^,278^**c**^,279^**s**^	[43]
*S*281	278^**c**^,279^**s**^	[43]
*Y*297	291^**c**^,295^**c**^,299^**c**^, 300^**s**^	[43]
*M*298	295^**c**^,299^**c**^,300^**s**^	[43]
*T*332	295^**c**^,299^**c**^	
*V*374	377^**c**^	
*A*378	377^**c**^,382^**s**^	[43]
*A*379	377^**c**^,382^**s**^	[43]
*S*383	382^**s**^,385^**s**^	[43]
*A*384	382^**s**^,385^**s**^	[43]
*A*387	385^**s**^	[43]
*S*388	385^**s**^,392^**s**^	[38,43]
*V*412	226^**s**^,227^**c**^,410^**c**^,414^**s**^,416^**s**^	[40,43]
*F*419	416^**s**^	[40]
*E*443	444^**c**^,445^**c**^,447^**s**^	[39]
*G*446	444^**c**^,445^**c**^,447^**s**^,448^**c**^,449^**c**^	[39]

^**s**^: non-synonymous snp site, ^**c**^: strictly conserved site.

In total, we have demonstrated here that according to the essential sites of GCK, 62 out of 64 QCMF- significant sites are functionally or structurally important for human GCK protein. The remaining two significant residue sites V89 and N283 do not overlap with essential sites and the reason for their significance and their role in the GCK protein is still unclear.

### Individual residue site comparison between QCMF-significant sites and previous CMF-significant sites

We compared QCMF-significant residue sites for both human EGFR and GCK proteins with the significant residue sites given in [5] of the previous CMF-method. The CMF-method detected for both human proteins, 43 sites in EGFR and 72 sites in GCK as significant.

For the EGFR protein we found that the QCMF-significant residue sites Q791, Q820, G824, K860, Y891, T892, Y900, T909 overlap with results of the CMF-method. Interestingly, one of the unconfirmed residue sites, the Q820, has been predicted by both QCMF-method and CMF-method as significant.

For GCK protein, we observed that in total 24 QCMF- significant sites (T60, T82, N83, F123, F148, T149, F152, H156, F171, N180, T206, T209, T228, E236, G260, L271, S281, N283, Q287, G294, E300, T332, F419 and E443) were also determined by the CMF-method as significant. Although both methods detected residue site N283 as significant, it corresponds to one of the unconfirmed residue sites for GCK, currently.

The CMF has been developed using normalized mutual information (MI) measures in order to detect important residue positions in MSAs. The method mainly focuses on significant BLOSUM62-dissimilar amino acid signals as a model of compensatory mutations and integrates them in the calculation of normalized MI-metrics. As a consequence of mainly taking into account dissimilar amino acid signals, an important part of CMF-significant sites were verified as disease associated nsSNP positions and just a small part of them were located at or near the catalytic sites, allosteric sites and binding sites in both proteins.

Moreover, when statistically evaluating both methods, we have observed that the QCMF significantly outperforms the QCMF-method. The QCMF reaches an improved performance in identifying essential sites from MSAs of both proteins with a significantly higher Matthews correlation coefficient (MCC) value of 0.215 whereas the CMF reaches only a MCC value of 0.133.

### Significant residue pair comparison

To analyze whether the quantum-information-theory-based measures proposed in this study complements the coventional methods for the detection of correlated (co-evolutionary) mutations, we made pairwise comparisons between our new QCMF, MIp [6], PSICOV [18], and CMF [5].

All four methods take as input an MSA satisfying certain admissibility criteria. The problem is that QCMF and CMF output the set of QCMF-significant sites and CMF-significant sites of *M*’s reference protein, respectively, whereas PSICOV and MIp result in sets of important residue pairs. To make these outputs comparable, we extend them in all cases.

Let $V_{\text{QCMF}}$ denote the output of QCMF on any admissible MSA *M*. We extend this set to what we call the QCMF-significant residue network $N_{\text{QCMF}}:=\left(V_{\text{QCMF}},E_{\text{QCMF}}\right)$ of *M* as follows. Any two elements of $V_{\text{QCMF}}$ are connected by an undirected edge belonging to $E_{\text{QCMF}}$ if and only if the corresponding column pair is QCMF-significant.

The CMF-significant residue network $N_{\text{CMF}}$ is analogously defined.

In order to get a sufficiently large number MIp-significant and PSICOV-significant residue pairs, for every input MSA we simply took the top-ranking 10% as MIp-significant and PSICOV-significant, respectively.

We then utilized the connectivity degree technique in the same way as we did for CMF and QCMF to calculate the set of MIp-significant sites $V_{\text{MIp}}$ and the set of PSICOV-significant sites $V_{\text{PSICOV}}$.

For all four methods we used the 90th, the 95th and the 99th percentile as *cut-off* values.

Finally, the edge sets $E_{\text{MIp}}$ and $E_{\text{PSICOV}}$ were determined by full analogy with the calculation of $E_{\text{QCMF}}$ and $E_{\text{CMF}}$. Thus we obtained the MIp-significant residue network $N_{\text{MIp}}$ and the PSICOV-significant residue network $N_{\text{PSICOV}}$.

We performed the method comparison edge-oriented, with the number of overlapping edges as measure. We applied all four methods to the 153 MSAs (see Additional files 1) described at the very beginning of this section and calculated the numbers $\left|E_{\text{QCMF}}^{\left(i\right)}\right|$, $\left|E_{\text{CMF}}^{\left(i\right)}\right|$, $\left|E_{\text{PSICOV}}^{\left(i\right)}\right|$, $\left|E_{\text{MIp}}^{\left(i\right)}\right|$, $\left|E_{\text{QCMF}}^{\left(i\right)}\cap E_{\text{MIp}}^{\left(i\right)}\right|$, $\left|E_{\text{QCMF}}^{\left(i\right)}\cap E_{\text{PSICOV}}^{\left(i\right)}\right|$, $\left|E_{\text{QCMF}}^{\left(i\right)}\cap E_{\text{CMF}}^{\left(i\right)}\right|$, $\left|E_{\text{MIp}}^{\left(i\right)}\cap E_{\text{PSICOV}}^{\left(i\right)}\right|$, $\left|E_{\text{MIp}}^{\left(i\right)}\cap E_{\text{CMF}}^{\left(i\right)}\right|$ and $\left|E_{\text{PSICOV}}^{\left(i\right)}\cap E_{\text{CMF}}^{\left(i\right)}\right|$ on each of them, where the connectivity cut-off ranges over the 90th, the 95th and the 99th percentile, and *i*=1,2,…,153. Summing up the 153 numbers in each of these groups results in the numbers $\overset{153}{\underset{i=1}{\sum }}\left|E_{\text{QCMF}}^{\left(i\right)}\right|$, $\overset{153}{\underset{i=1}{\sum }}\left|E_{\text{CMF}}^{\left(i\right)}\right|$, $\overset{153}{\underset{i=1}{\sum }}\left|E_{\text{PSICOV}}^{\left(i\right)}\right|$, $\overset{153}{\underset{i=1}{\sum }}\left|E_{\text{MIp}}^{\left(i\right)}\right|$, $\overset{153}{\underset{i=1}{\sum }}\left|E_{\text{QCMF}}^{\left(i\right)}\cap E_{\text{MIp}}^{\left(i\right)}\right|$, $\overset{153}{\underset{i=1}{\sum }}\left|E_{\text{QCMF}}^{\left(i\right)}\cap E_{\text{PSICOV}}^{\left(i\right)}\right|$, $\overset{153}{\underset{i=1}{\sum }}\left|E_{\text{QCMF}}^{\left(i\right)}\cap E_{\text{CMF}}^{\left(i\right)}\right|$, $\overset{153}{\underset{i=1}{\sum }}\left|E_{\text{MIp}}^{\left(i\right)}\cap E_{\text{PSICOV}}^{\left(i\right)}\right|$, $\overset{153}{\underset{i=1}{\sum }}\left|E_{\text{MIp}}^{\left(i\right)}\cap E_{\text{CMF}}^{\left(i\right)}\right|$ and $\overset{153}{\underset{i=1}{\sum }}\left|E_{\text{PSICOV}}^{\left(i\right)}\cap E_{\text{CMF}}^{\left(i\right)}\right|$, which are displayed in Tables 3 and 4.

**Table 3:** Total number of edges in method-dependent significant residue networks with respect to various connectivity degree cut-offs

	**Total number of edges in significant residue networks**		
**Connectivy degree cut-off**	**90 *% *** **th percentile**	**95 *% *** **th percentile**	**99 *% *** **th percentile**
$\overset{153}{\underset{i=1}{\sum }}\left|E_{\text{QCMF}}^{\left(i\right)}\right|$	82561	20411	435
$\overset{153}{\underset{i=1}{\sum }}\left|E_{\text{MIp}}^{\left(i\right)}\right|$	90636	24094	1454
$\overset{153}{\underset{i=1}{\sum }}\left|E_{\text{PSICOV}}^{\left(i\right)}\right|$	80489	21596	1088
$\overset{153}{\underset{i=1}{\sum }}\left|E_{\text{CMF}}^{\left(i\right)}\right|$	87208	23893	936

**Table 4:** Total number of edges in two networks of different type with respect to various connectivity degree cut-offs

	**Total number of common edges in two networks of different type**		
**Connectivy degree cut-off**	**90 *% *** **th percentile**	**95 *% *** **th percentile**	**99 *% *** **th percentile**
$\overset{153}{\underset{i=1}{\sum }}\left|E_{\text{QCMF}}^{\left(i\right)}\cap E_{\text{MIp}}^{\left(i\right)}\right|$	898	77	0
$\overset{153}{\underset{i=1}{\sum }}\left|E_{\text{QCMF}}^{\left(i\right)}\cap E_{\text{PSICOV}}^{\left(i\right)}\right|$	735	64	0
$\overset{153}{\underset{i=1}{\sum }}\left|E_{\text{QCMF}}^{\left(i\right)}\cap E_{\text{CMF}}^{\left(i\right)}\right|$	4036	474	1
$\overset{153}{\underset{i=1}{\sum }}\left|E_{\text{MIp}}^{\left(i\right)}\cap E_{\text{PSICOV}}^{\left(i\right)}\right|$	9094	1488	11
$\overset{153}{\underset{i=1}{\sum }}\left|E_{\text{MIp}}^{\left(i\right)}\cap E_{\text{CMF}}^{\left(i\right)}\right|$	3343	474	6
$\overset{153}{\underset{i=1}{\sum }}\left|E_{\text{PSICOV}}^{\left(i\right)}\cap E_{\text{CMF}}^{\left(i\right)}\right|$	2618	368	2

Table 3 shows that all methods detect with the same connectivity degree cut-off a comparable number of edges in the corresponding significant residue network.

Table 4 highly suggests that all four methods carry distinct information. The overlap between any two of them is less than or equal to 10%. This indicates that, under the assumption that each of them models important aspects of co-evolution, they complement each other perfectly. In particular, this is true for QCMF as a quantum-information-science-based service compared with the other three established tools that are based on conventional methods.

### Implementation of QCMF: Parallel computing using CUDA

The computation of both QCMF metrics (Equations 7 and 8) is strongly based on matrix operations. Therefore, we implement QCMF algorithm using CUDA [25] which is very suitable to perform large number of vector and matrix operations in real time. This results in a dramatic reduction of computational time of QCMF.

**Figure 5:**
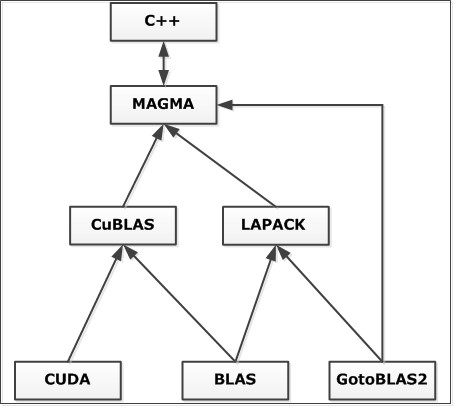
Linking of the CUDA environment using C++.

In this study, we use the CUDA 4.0 architecture (Toolkit) with several linear algebra libraries such as MAGMA [52], LAPACK [53], BLAS [54], GotoBLAS [55], CUBLAS [25] together (see Figure 5) to speed up the running time of the QCMF algorithm. Since our program requires a cooperative multi threading to not fall in any asynchronicity or locks we extended the magma library with dynamic scheduling features according to [56]. Further, in order to be able to compare the performance, we also implemented the QCMF algorithm onto CPU architecture alone. Both implementations were performed on an Intel Core™ i7-3770K Processor operating at 3.9GHz, with 16 GB of DDR3 RAM and a GeForce GTX 680 graphics card using the Ubuntu 13.04 operating system (64-bit version).

Applying the QCMF algorithm for human EGFR protein with CPU alone and with CUDA acceleration, the average computational time of a column pair was 0.7117 seconds and 0.0301 seconds, respectively. Similarly, for human GCK protein, the average computational time of a column pair was 0.6977 seconds with CPU alone and 0.0299 seconds with CUDA acceleration. Consequently, the algorithm took ∼310 minutes for human EGFR protein and ∼811 minutes for GCK protein with CPU alone. On the other hand, applying the CUDA acceleration it took only ∼13 minutes for EGFR and ∼39 minutes for GCK protein. The comparison between the average times indicates that the required computational time of QCMFalgorithm with the CUDA acceleration was significantly faster than with CPU alone (approximately more than 23 times faster).

## Methods

We predict important sites of a protein by detecting co-evolving residues. Our measures of co-evolution are quantum-Jensen-Shannon-divergence-based metrics of column pairs of a multiple sequence alignment, with the protein under study being the reference row. The quantum Jensen-Shannon divergence in turn has the von Neumann entropy as main building block.

The von Neumann entropy was originally defined in the framework of quantum mechanics. We elucidate it in the subsequent section as far as it is necessary to understand our methods. Researchers interested in learning more are referred to the excellent textbook due to Vedral [57]. A comprehensive reference book was published by Nielsen and Chuang [58].

This section is organized as follows. In the first four subsections we recapitulate techniques developed in [5] which we leverage in this study. This concerns the definition of significant site pairs and of significant individual sites, the preparation of the training data set used, and the computation of a doubly stochastic matrix *D* as our model of compensatory mutations on grounds of two counting matrices *C*_alt_ and *C*_null_. These two matrices also form the basis of the two amino acid pair similarity matrices $A_{\text{ent}}$ and $A_{\text{sep}}$, which in turn give rise to our new quantum-information-science-based metrics ${\mathbb{Q}}_{\text{ent}}$ and ${\mathbb{Q}}_{\text{sep}}$. The last four subsection are dedicated to their definitions.

### Significant column pairs and significant position with respect to a certain metric

Let *M* be an MSA, where the protein of interest is represented by *M*’s first row, and let  be a metric which assigns to every MSA column pair (*γ*_1_,*γ*_2_) a real number $E\left(\gamma_{1},\gamma_{2}\right)\in [0,1]$. We call  a *co-evolutionary column pair metric* if it models a biologically meaningful co-evolutionary signal: The larger the metric value on (*γ*_1_,*γ*_2_), the more likely co-evolution between position *γ*_1_ and position *γ*_2_ has occurred.

Let ${\hat{p}}_{\left(i,j\right)}$ be the empirical relative amino acid pair frequency of the *i*-th and the *j*-th amino acid in column pair (*γ*_1_,*γ*_2_), where *i*,*j*=1,2,…,20. (When choosing a row of this column pair by pure chance, acid pair (*i*,*j*) is drawn with probability ${\hat{p}}_{\left(i,j\right)}$.) In the subsequent subsection we recapitulate the way developed in [5] to identify significant columns and significant column pairs with respect to .

A well-studied example (see [5,12]) of a co-evolutionary column pair metric is the normalized mutual information

(1) $$U(\gamma_{1},\gamma_{2}):=2\cdot \frac{ℍ(\gamma_{1})+ℍ(\gamma_{2})-ℍ(\gamma_{1},\gamma_{2})}{ℍ(\gamma_{2}+ℍ\gamma_{2})},$$

where $ℍ(\gamma_{1},\gamma_{2})$, $ℍ(\gamma_{1})$, and $ℍ(\gamma_{2})$ denote the Shannon entropy of the empirical pair distribution ${\left({\hat{p}}_{\left(i,j\right)}\right)}_{i,j=1,2,\ldots ,20}$ of the column pair (*γ*_1_,*γ*_2_) and its two marginals.

In order to identify significant column pairs of the MSA under study with respect to the metric , in [5] we have pointed out, that the distribution of  can be regarded as a mixture of a background *β*-distribution *F*_0_, an unrelated pair distribution *G*_1_, and a distribution *G*_2_ of presumably co-evolving pairs.

The *p*-values $1-F_{0}\left(E\right)$ are then uniformly distributed over [ 0,1] given the underlying -values are *F*_0_-distributed. In contrast, *p*-values tend to zero or one, if -values are *G*_2_-distributed or *G*_1_-distributed, respectively.

If, moreover, there is a sub-interval of [ 0,1] which contains only data from the background distribution, on grounds of a result due to Storey and Tibshirani [59,60] we determined in [5] an MSA-dependent threshold for -values. A column pair is said to be (E,M)-significant, if its -value is above the threshold, where the false discovery rate is bounded by a predefined constant.

**Figure 6:**
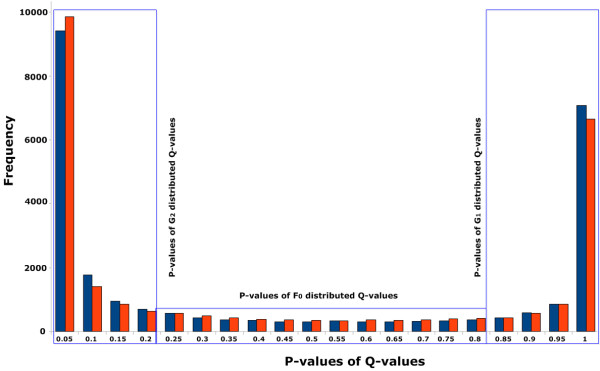
**p-value distributions of**${\mathbb{Q}}_{\text{ent}}$** and**${\mathbb{Q}}_{\text{sep}}$**-values for human EGFR protein (PDB-Entry 2J6M).** The blue bars illustrate the ***p***-value distribution of the ${\mathbb{Q}}_{\text{ent}}$-values and red bars display the *p*-value distribution of the ${\mathbb{Q}}_{\text{sep}}$-values.

Figure 6 is a typical pictorial representation of metric distributions which can be treated that way to detect significant pairs.

We applied that model in this study.

We utilized the connectivity degree technique, introduced in [12] and developed further in [5], in order to define the (E,M)-significance of individual residue sites. The connectivity degree of a position *γ*_1_ is the number of positions *γ*_2_ so that the site pair (*γ*_1_,*γ*_2_) is (E,M)-significant. A site of the protein of interest is then called (E,M)-significant, if its connectivity degree *cut-off* exceeds the 90-th percentile.

### Training data set and pre-processing

Following [5], a redundancy free set of more than 35000 protein structures is our starting point. This collection was compiled in Rainer Merkl’s Lab at the University of Regensburg. The protein structures were taken from the protein data base (http://www.pdb.org/). The PISCES services [61] was applied to assess proteins on sequence similarity and equality of 3D-data. The related MSAs were gathered from the HSSP data base (http://swift.cmbi.ru.nl/gv/hssp/).

Taking pattern from [12], we filtered every MSA obtained as follows. First, highly similar and dissimilar sequences were deleted to ensure that the sequence identity between any two sequences is at least 20% and no more than 90%. Second, we removed strictly conserved residue columns, where the percentage of identical residues is greater than 95%. Third, we eliminated the residue columns which contain more than 25% gaps. Finally, we discarded all MSAs with less than 125 sequences. More than 17000 MSAs survived the last filtering step. We used approximately 1700 MSAs published in [5] as our *training data set* which we randomly chose from this set.

### Setting up the counting matrices *C*_alt_ and *C*_null_

The entries of the two matrices are frequencies of pair substitutions calculated from our training data set described in the foregoing subsection. Informally spoken, matrix *C*_alt_ models the signal, whereas *C*_null_ reflects the background.

In line with [5], we calculated a signal and a null set of column pairs. The signal set consists of all (U,M)-significant column pairs, where *M* ranges over all training MSA. The null set consists of sufficiently many column pairs randomly chosen from every training MSA. For both the signal set and the null set we computed a symmetric 400×400 integer-valued matrix of frequencies of pair substitutions *C*_alt_ and *C*_null_. To this end, the method used to compute BLOSUM62 matrices [62] is applied to count residue pair substitutions in MSA column pairs rather than residue substitution in columns.

### Computing a doubly stochastic matrix *D*

According to [5], a pair ((*a*_*i*_,*a*_*j*_),(*a*_*k*_,*a*_*l*_)) of amino acid pairs is said to be a *formal dissimilar compensatory mutation*, if the BLOSUM62 score both of (*a*_*i*_,*a*_*k*_) and (*a*_*j*_,*a*_*l*_) is negative.

Using *C*_alt_ and *C*_null_, we define the matrix *C*_CompMut_ by

() $$\begin{array}{lll} & \;C_{\text{CompMut}}\left(\left(a_{i},a_{j}),(a_{k},a_{l}\right)\right)\; & \;\\ & \;\;\;\;:=\left\{\begin{array}{l} C_{\text{alt}}\left(\left(a_{i},a_{j}),(a_{k},a_{l}\right)\right)\;\;\text{if}\;\;\varphi_{\text{CompMut}}\left(\left(a_{i},a_{j}),(a_{k},a_{l}\right)\right)=1;\\ 0\;\text{otherwise}; \end{array}\right)\; & \; \end{array}$$

where *φ*_CompMut_((*a*_*i*_,*a*_*j*_),(*a*_*k*_,*a*_*l*_))=1 if and only if either (*a*_*i*_,*a*_*j*_)=(*a*_*k*_,*a*_*l*_) or ((*a*_*i*_,*a*_*j*_),(*a*_*k*_,*a*_*l*_)) is a formal dissimilar compensatory mutation and

$$\begin{array}{lll} & \frac{C_{\text{alt}}\left(\left(a_{i},a_{j}),(a_{k},a_{l}\right)\right)}{\underset{i^{\prime },j^{\prime },k^{\prime },l^{\prime }}{\sum }C_{\text{alt}}\left(\left(a_{i^{\prime }},a_{j^{\prime }}),(a_{k^{\prime }},a_{l^{\prime }}\right)\right)}\; & \;\\ & >\frac{C_{\text{null}}\left(\left(a_{i},a_{j}),(a_{k},a_{l}\right)\right)}{\underset{i^{\prime },j^{\prime },k^{\prime },l^{\prime }}{\sum }C_{\text{null}}\left(\left(a_{i^{\prime }},a_{j^{\prime }}),(a_{k^{\prime }},a_{l^{\prime }}\right)\right)}.\; & \; \end{array}$$

By normalizing *C*_CompMut_, we obtain a symmetric matrix *P*_CompMut_. For *a*_*i*_,*a*_*j*_,*a*_*k*_,*a*_*l*_ ranging over all amino acids, *P*_CompMut_((*a*_*i*_,*a*_*j*_),(*a*_*k*_,*a*_*l*_)) represents an empirical probability distribution on pairs of amino acid pairs.

We then calculated the symmetric 400×400-matrix

$$S_{\text{CompMut}}:={\left(\log\frac{P_{\text{CompMut}}\left(\left(a_{i},a_{j}),(a_{k},a_{l}\right)\right)}{P_{\text{CompMut}}^{b}\left(a_{i},a_{j}\right)P_{\text{CompMut}}^{b}\left(a_{k},a_{l}\right)}\right)}_{\left(a_{i},a_{j}),(a_{k},a_{l}\right)},$$

where $P_{\text{CompMut}}^{b}\left(a_{i},a_{j}\right)$ is the marginal distribution of *P*_CompMut_.

Having set all negative entries of *S*_CompMut_ to zero, the doubly stochastic matrix *D* is computed by means of the canonical iterated row-column normalization procedure [63].

The doubly stochastic *D* is used to linearly transform empirical amino acid pair distributions of column pairs. If the pair distribution is regarded as a 400-dimensional row vector, matrix *D* is multiplied from the right. If then, for example, the resulting distribution is plugged into Equation 1, column pairs containing formal dissimilar compensatory mutations the *D*-transition probability of which is relatively large tend to be up-scaled.

The idea of the subsequent subsections is to design a model of MSA column pairs that takes formal dissimilar compensatory mutations regarded as pair dissimilarities as well as pair similarities into account. The challenge is to implement this in a way such that these two effects interfere but do not interact. This is necessary since a similarity relation is transitive, whereas a dissimilarity relation is not.

### Setting up the two counting matrices *C*_ent_ and *C*_sep_

We set up two significant pair substitution matrices *C*_ent_ and *C*_sep_ from *C*_alt_ and *C*_null_ which form the basis of our new metrics ${\mathbb{Q}}_{\text{ent}}$ and ${\mathbb{Q}}_{\text{sep}}$. The intuition behind *C*_ent_ is that the component-wise BLOSUM62-based pair similarity is rescaled, whereas *C*_sep_ leads to a new amino acid pair similarity.

$$\begin{array}{lll} & \;C_{\text{ent}}\left(\left(a_{i},a_{j}),(a_{k},a_{l}\right)\right)\; & \;\\ & \;\;\;\;\;:=\;\left\{\begin{array}{l} C_{\text{alt}}\left(\left(a_{i},a_{j}),(a_{k},a_{l}\right)\right)\;\;\;\text{if}\;\;\varphi_{\text{ent}}\left(\left(a_{i},a_{j}),(a_{k},a_{l}\right)\right)\;=\;1;\\ 0\;\text{otherwise}; \end{array}\right)\; & \; \end{array}$$

where *φ*_ent_((*a*_*i*_,*a*_*j*_),(*a*_*k*_,*a*_*l*_))=1 if and only if either (*a*_*i*_,*a*_*j*_)=(*a*_*k*_,*a*_*l*_) or the following two conditions are satisfied. First, the amino acids *a*_*i*_ and *a*_*k*_ as well as the amino acids *a*_*j*_ and *a*_*l*_ are BLOSUM62-similar. Second,

(2) $$\begin{array}{lll} & \frac{C_{\text{alt}}\left(\left(a_{i},a_{j}),(a_{k},a_{l}\right)\right)}{\underset{i^{\prime },j^{\prime },k^{\prime },l^{\prime }}{\sum }C_{\text{alt}}\left(\left(a_{i^{\prime }},a_{j^{\prime }}),(a_{k^{\prime }},a_{l^{\prime }}\right)\right)}\; & \;\\ & >\frac{C_{\text{null}}\left(\left(a_{i},a_{j}),(a_{k},a_{l}\right)\right)}{\underset{i^{\prime },j^{\prime },k^{\prime },l^{\prime }}{\sum }C_{\text{null}}\left(\left(a_{i^{\prime }},a_{j^{\prime }}),(a_{k^{\prime }},a_{l^{\prime }}\right)\right)}.\; \end{array}$$

$$\begin{array}{lll} & \;C_{\text{sep}}\left(\left(a_{i},a_{j}),(a_{k},a_{l}\right)\right)\; & \;\\ & \;\;\;\;\;:=\;\left\{\begin{array}{l} C_{\text{alt}}\left(\left(a_{i},a_{j}),(a_{k},a_{l}\right)\right)\;\;\;\text{if}\;\;\varphi_{\text{sep}}\left(\left(a_{i},a_{j}),(a_{k},a_{l}\right)\right)\;=\;1;\\ 0\;\text{otherwise}; \end{array}\right)\; & \; \end{array}$$

where *φ*_sep_((*a*_*i*_,*a*_*j*_),(*a*_*k*_,*a*_*l*_))=1 if and only if either (*a*_*i*_,*a*_*j*_)=(*a*_*k*_,*a*_*l*_) or Equation 2 is satisfied.

### 

Calculating the two amino acid pair similarity matrices

$$A_{\text{ent}}$$

and

$$A_{\text{sep}}$$

Recall that a matrix *A* is positive definite (positive semi-definite), if there is an orthogonal matrix *U* (defining property *U*^−1^=*U*^*T*^) such that *UAU*^*T*^ is a diagonal matrix, where the coefficients in the main diagonal are strictly positive (non-negative).

Let us call a 400×400-matrix  a *amino acid pair similarity matrix*, if  is positive definite and the entries in the main diagonal are equal to 1, whereas the off-diagonal elements $A_{\left(g,h),(i,j\right)}$ ((*g*,*h*)≠(*i*,*j*)) are greater than or equal to 0, but less than 1.

The entries of an amino acid pair similarity matrix  are interpreted as follows. The closer $A_{\left(g,h),(i,j\right)}$ to 1, the more similar are the amino acid pairs (*g*,*h*) and (*i*,*j*).

Let *C* be either *C*_ent_ or *C*_sep_. We define

$$B_{\left(g,h),(i,j\right)}:=\frac{C_{\left(g,h),(i,j\right)}^{\alpha }}{\sqrt{\overset{20}{\underset{\iota ,\kappa =1}{\sum }}C_{\left(\iota ,\kappa ),(i,j\right)}^{2\alpha }}},$$

where ((*g*,*h*),(*i*,*j*)) ranges over all possible 160000 indices of pairs of amino acid pairs including the main diagonal, and *α*∈(0,1) was set to 0.1 in order to enhance the effect of similarity.

Because of the fact, that matrix *B* is not in any case positive definite, we finally set

(3) $$A:=B^{T}B,$$

which is justified by the transitivity of similarity. That way the amino acid similarity matrices $A_{\text{ent}}$ and $A_{\text{sep}}$ are obtained from the counting matrices *C*_ent_ and *C*_sep_, respectively.

Amino acid pair similarity matrices generalize amino acid similarity matrices used by Johansson et al. [24] for evaluating amino acid conservation.

### Modeling MSA column pairs and single columns by means of density matrices

Let (*γ*_1_,*γ*_2_) be a column pair of a multiple sequence alignment, let ${\left({\hat{p}}_{\left(i,j\right)}\right)}_{i,j=1,2,\ldots ,20}$ be the empirical amino acid pair distribution in these columns, let ${\left({\hat{q}}_{\left(i,j\right)}\right)}_{i,j=1,2,\ldots ,20}$ be the linear transform of ${\left({\hat{p}}_{\left(i,j\right)}\right)}_{i,j=1,2,\ldots ,20}$ by the doubly stochastic matrix *D*, and let  be an amino acid pair similarity matrix.

Recall, that the trace of a matrix is the sum of its coefficients in the main diagonal.

Taking pattern from quantum mechanics, we model column pair (*γ*_1_,*γ*_2_) by a positive semi-definite 400×400-matrix the trace of which is equal to 1, a so-called *density matrix*. Regarding the two distributions ${\left({\hat{p}}_{\left(i,j\right)}\right)}_{i,j\;=\;1,2,\ldots ,20}$ and ${\left({\hat{q}}_{\left(i,j\right)}\right)}_{i,j\;=\;1,2,\ldots ,20}$ as 400×400-diagonal matrices the main diagonal of which are formed by the probabilities ${\hat{p}}_{\left(i,j\right)}$ and ${\hat{q}}_{\left(i,j\right)}$, respectively, we integrate the classical model into the quantum-mechanics-based one.

Generalizing the approach for amino acid used in [24] to amino acid pairs, our density matrices are of the shape

(4) $$\rho \left(\hat{r},A\right):={\left(\sqrt{{\hat{r}}_{\left(g,h\right)}}A_{\left(g,h),(i,j\right)}\sqrt{{\hat{r}}_{\left(i,j\right)}}\right)}_{g,h,i,j\;=\;1,2,\ldots ,20},$$

where ${\hat{r}}_{\left(i,j\right)}$ is either ${\hat{p}}_{\left(i,j\right)}$ or ${\hat{q}}_{\left(i,j\right)}$ (*i*,*j*=1,2,…,20). Using this denotation, the diagonal density matrices considered in the preceding paragraph are equal to some $\rho \left(\hat{r},1\right)$, where 1 is the 400×400-identity matrix.

In this study, we regard individual MSA columns only as components of column pairs. In the classical case, where MSA-column pair (*γ*_1_,*γ*_2_) is modeled by an MSA-dependent amino acid pair distribution $\hat{r}$ (either ${\left({\hat{p}}_{\left(i,j\right)}\right)}_{i,j\;=\;1,2,\ldots ,20}$ or some derivative), the columns *γ*_1_ and *γ*_2_ are represented by the corresponding marginals ${\hat{r}}_{1}$ and ${\hat{r}}_{2}$ of $\hat{r}$.

In quantum information science, the counter part of the marginals ${\hat{r}}_{1}$ and ${\hat{r}}_{2}$ of $\hat{r}$ are the partial traces tr_2_(*ρ*) and tr_1_(*ρ*) of *ρ*. They are 20×20 density matrices defined by

$${\left({\text{tr}}_{1}(\rho )\right)}_{\text{ij}}:=\overset{20}{\underset{k=1}{\sum }}\rho_{\text{kkij}}\;{\left({\text{tr}}_{2}(\rho )\right)}_{\text{ij}}:=\overset{20}{\underset{k=1}{\sum }}\rho_{\text{ijkk}},$$

where *i*,*j*=1,2…,20. As opposed to the indices of the marginals, matrix tr_1_(*ρ*) models column *γ*_2_, whereas matrix tr_2_(*ρ*) represents column *γ*_1_.

### Defining our two new metrics ${\mathbb{Q}}_{\text{ent}}$ and ${\mathbb{Q}}_{\text{sep}}$

To begin with, we define the von Neumann entropy VNE(*ρ*) of a diagonal density matrix *ρ* as the Shannon entropy of its main diagonal coefficients regarded as a probability distribution.

The crucial property of a density matrix *ρ* is that there exists an orthogonal matrix *U* such that *UρU*^*T*^ is a diagonal density matrix, where the diagonal elements are uniquely determined up to their order. Thus we are justified to finally define

(5) $$\text{VNE}\left(\rho \right):=\text{VNE}\left(\mathrm{U\rho}U^{T}\right),$$

where *U* is an orthogonal matrix diagonalizing *ρ* in a way just mentioned.

In principle, the following holds true. The larger the off-diagonal coefficients of the similarity matrix , the smaller the von Neumann entropy of the density matrix according to Equation 4 compared with the Shannon entropy of the probability distribution ${\hat{r}}_{\left(i,j\right)}$ (*i*,*j*=1,2,…,20).

In order to compare two density matrices *ρ* and *σ* of the same dimension, we make use of the quantum Jensen-Shannon divergence:

(6) $$\text{QJSD}(\rho ∥\sigma ):=\;\text{VNE}\left((\rho +\sigma )/2\right)-\left(\text{VNE}(\rho )\;+\;\text{VNE}(\sigma )\right)/2.$$

It can be shown that 0≤QJSD(*ρ*∥*σ*)≤1, where 0 is attained if and only if the two density matrices *ρ* and *σ* are equal. As oppose to the case of Equation 1, we have thus avoided a normalization.

We are now in a position to define our new two metrics for a certain column pair of a given MSA. As before, the amino acid pair distribution $\hat{q}$ is given by $\hat{p}\cdot D$, where *D* is the 400×400 doubly stochastic matrix described above, $\hat{p}$ is the empirical pair distribution of these two columns, and 1 is the 400×400-identity matrix.

Then our first metric ${\mathbb{Q}}_{\text{ent}}$ is defined by

(7) $${\mathbb{Q}}_{\text{ent}}:=\text{QJSD}\left(\rho \left(\hat{q},A_{\text{ent}}\right)∥\rho \left(\hat{p},1\right)\right)$$

(see Equation 4). This metric measures the difference between a density matrix combining rescaled amino acid pair similarity with dissimilar compensatory mutations and the empirical amino acid pair distribution. The index “*ent*” indicates that here we make use of quantum entanglement, which in turn is a major resource of quantum information science. (Entangled 400×400-density matrices are those that cannot be represented as a convex combination of Kronecker products of 20×20-density matrices. Note, that the Kronecker product of density matrices is the analog of the classical product of probability distributions).

Our second new metric ${\mathbb{Q}}_{\text{sep}}$ is given by

(8) $${\mathbb{Q}}_{\text{sep}}:=\text{QJSD}\left({\text{tr}}_{1}\left(\rho \left(\hat{p},A_{\text{sep}}\right)\right)∥{\text{tr}}_{2}\left(\rho \left(\hat{p},A_{\text{sep}}\right)\right)\right).$$

The density operator $\rho \left(\hat{p},A_{\text{sep}}\right)$ is entangled. However, before finally calculating the metric, we separate the columns of the pair by applying the two partial trace operators.

Using the example of the human EGFR protein (PDB-Entry 2J6M), Figure 6 illustrates that the method we developed in [5] to determine significant column pairs is well-applicable for both ${\mathbb{Q}}_{\text{ent}}$ and ${\mathbb{Q}}_{\text{sep}}$. The results presented in this work prove that ${\mathbb{Q}}_{\text{ent}}$ as well as ${\mathbb{Q}}_{\text{sep}}$ are powerful co-evolutionary column pair metrics.

## Discussion

Grosse *et al.* observed in [64] that the Jensen-Shannon divergence (JSD) can be interpreted as mutual information between two (or more) random sources in a special setting particularly appropriate to discriminate between these sources. This is what we need when it comes to predicting important protein sites in an MSA-based approach. It might explain the findings of Capra and Singh [22] on the predictive power of JSD. These two articles encouraged us to utilize quantum Jensen-Shannon divergence (QJSD) in this study. As a side effect, a normalization is not necessary, since quantum Jensen-Shannon divergence, like its classical counterpart, ranges over the real interval [ 0,1].

Several studies have confirmed the fact that detecting coupled MSA-columns is extremely useful in the prediction of important protein sites (see e.g. [4-6,10-13,65-70]). When using information-theoretic metrics, there is no doubt that it is reasonable to incorporate amino acid pair dissimilarity as well as amino acid similarity in a consistent way such that similarity decreases entropy, whereas dissimilarity increases it. This kind of consistency is important, since entropy is the fundamental building block for most of those metrics. In particular, the Jensen-Shannon divergence between two probability mass functions (pmfs) *p* and *q* equals ℍ(1/2(p+q))−1/2(ℍ(p)+ℍ(q)).

In [5] an amino acid pair dissimilarity model for compensatory mutations is presented. A doubly stochastic matrix transforms the empirical amino acid pair distribution of a column pair.

Rescaled pair similarity of BLOSUM62-similar pairs is to capture an aspect of coupled MSA column pairs orthogonal to the phenomenon of dissimilar compensatory mutations. It models the amino acid pair transition preferences within those column pairs on the average. As suggested by Caffrey *et al.*[23] as well as Johansson *et al.*[24], it is promising to incorporate them within the framework of quantum information theory. Therein, density matrices replace pmfs. The counterpart of the entropy of a pmf is the von Neumann entropy (VNE) of a density matrix (see Equation 5). QJSD corresponds then exactly to JSD (see Equation 6).

The challenge was to complement the model presented in [5] by additionally incorporating amino acid pair similarity in a way that the two effects interfere but do not interact. We model an MSA column pair by means of a 400×400-density matrix, rather than amino acid pair distributions. This provides us with the opportunity to utilize the notion of entanglement, which in turn is a major resource of quantum information. In our model, partial traces play the role of the marginals in the classical case. Pair similarity is reflected by means of positive definite pair similarity matrices (see Equation 3), where positive definiteness, which is a key property of density matrices, can only be ensured by using transitivity of similarity. Since there is no transitivity of dissimilarity, we kept dissimilarity apart from that similarity matrix. Instead, we carried over the CMF dissimilarity model of [5]. Similarity matrix and transformed amino acid pair distribution are joined together by means of Equation 4 in the final step of our density matrix design. That way we minimize the interaction between the two effects of dissimilarity and similarity.

In order to eliminate the noise and to define an MSA-dependent threshold for significant column pairs, we followed the line of [5]. The model presented there seems to be of universal applicability. The same is true for the connectivity degree model introduced in [12] and further developed in [5]. Combining them results in a reliable and robust method to determine significant residues.

The results we present in this study show that the vast majority of QCMF-significant residue sites are closely related to functionality and structural stability of both human EGFR and GCK proteins. 10 significant residue sites in EGFR and 19 significant sites in GCK are established as functionally important since they are directly located at or close to catalytic sites, allosteric sites and binding sites which are crucial for maintaining protein functions and for understanding the underlying molecular mechanism (see Figures 1,2,3,4). Additionally, 2 significant sites in EGFR and 8 significant sites in GCK (three of them are also in contact with allosteric sites in GCK) are related to disease associated nsSNP regions of both proteins. As has been noted in [5], most disease-causing mutations at these positions in corresponding sequences destroy structural features of proteins, thus affecting protein stability and often results in loss of protein function.

Although the importance of almost all QCMF-significant sites are verified through essential sites of both human proteins, there are still eight and two unconfirmed significant sites in EGFR and GCK proteins, respectively, which do not fall into essential sites. It is interesting to note that some of these unconfirmed sites are also referred as significant by CMF [5]. We therefore believe that most of these unconfirmed sites identified by our present method may have an importance for the function and structural stability of both proteins notwithstanding the absence of previous experimental data. A further comparison reveals that the overlaps between the results of the QCMF method and the CMF method are quite low, indicating that both methods detect considerably different sets of residue sites as functionally and structurally important. The comparison results clearly show that considering similar and dissimilar amino acid signals simultaneously, our present method is more sensible to catalytic, allosteric and binding sites, while only focusing on dissimilar signals the previous method deals successfully with nsSNP positions in proteins.

The final comparison between QCMF and CMF on EGFR and GCK proteins is made by inspecting several connectivity degree cut-offs. We initially set it to the 90-th percentile at which CMF reaches its maximal MCC value. Going through all possible *n*-th percentiles for *n*=80,81,…,99, QCMF reaches its maximal MCC value of 0.231 if *n*=88. What we got can be summarized as follows. On the one hand QCMF shows a better performance than CMF in identifying important residue sites. On the other hand QCMF complements CMF. This is because of the fact that the method of QCMF is more information rich than that of CMF. QCMF simultaneously uses similar and dissimilar amino acid pair signals, whereas CMF’s method focuses only on amino acid pair dissimilarity.

To confirm the educated guess that QCMF complements conventional methods both from information theory and statistics, we applied QCMF, CMF [5], MIp [6] and PSICOV [18] to the 153 MSAs described at the beginning of the Results section. In sum, each of these methods detects different residue pairs as important, where the pairwise overlap is bounded from above by 10%. The reason for that is that the four methods model different aspects of amino acid pair co-evolution. Consequently, they carry distinct information.

To further improve the specificity of QCMF it is promising to combine its quantum-information-theory-based framework with the direct pair distribution derived in DCA (see e.g. [15] or [16]).

## Conclusions

In this work, we report a new method, QCMF, applying principles of quantum information theory. In contrast to the previous method CMF which focused on dissimilar amino acid signals, QCMF simultaneously models similar and dissimilar amino acid pair signals in the detection of functionally or structurally important sites. QCMF includes two metrics based on quantum Jensen-Shannon divergence. While the first metric measures compensatory mutations between pairs of columns, the second metric considers the sequence conservation of columns. Results show that QCMF reaches an improved performance in identifying important sites from MSAs and it predicts a quite different set of residue sites as functionally and structurally important (in comparison to the previous method). Further, results indicate that the residue sites found by QCMF are more sensible to catalytic sites, allosteric sites and binding sites than those found by the previous method. On the top of that, a pairwise comparison with existing methods shows that QCMF is complementary to them when it comes to predicting co-evolving residue site pairs.

## Competing interests

The authors declare that they have no competing interests.

## Authors’ contributions

SW developed the model underlying QCMF. EW adjusted the model together with SW and interpreted the results. MG developed the model together with SW, designed and implemented the tool together with SH, SJJ and GD, and interpreted the results together with EW and CM. SH designed and carried out the CUDA programming. GD and SJJ supported SW in developing the model and MG in designing and implementing the tool. CM interpreted the results together with EW and MG. All authors read and approved the manuscript.

## Supplementary Material

Additional file 1 **Pdb entries.** Pdb entries of 153 test proteins. Click here for file

Additional file 2 **EGFR significant sites.** QCMF-significant residue sites of the human epidermal growth factor receptor (EGFR) protein. Click here for file

Additional file 3 **GCK significant sites.** QCMF-significant residue sites of the human glucokinase (GCK) protein. Click here for file
